# Bimaxillary Keratocystic Odontogenic Tumour: A Case of Diagnostic and Therapeutic Difficulty

**DOI:** 10.1155/2014/194810

**Published:** 2014-03-26

**Authors:** Victoria Nwebuni Okoje-Adesomoju, Akinyele Olumuyiwa Adisa, Olalere Omoyosola Gbolahan, Mofoluwaso Abimbola Olajide

**Affiliations:** ^1^Department of Oral and Maxillofacial Surgery, University College Hospital, Ibadan 200009, Nigeria; ^2^Department of Oral Pathology, University College Hospital, Ibadan 200009, Nigeria

## Abstract

Keratocystic odontogenic tumour (KCOT) is a benign cystic intraosseous tumour of odontogenic origin that is usually solitary except when syndromic. It rarely occurs in the maxilla; therefore a rapidly progressive, nonsyndromic bimaxillary KCOT with locoregional extension poses significant diagnostic and management challenges. To the best of the authors' knowledge, documentation of a nonsyndromic bimaxillary KCOT is nonexistent in the English literature. We therefore present the case of an extensive bimaxillary KCOT in a 38-year-old Nigerian male.

## 1. Introduction

Phillipsen was the first to report the entity odontogenic keratocyst (OKC) in 1956 [1]. Unlike other benign odontogenic cysts, OKC tends to be clinically aggressive, demonstrating a high mitotic count and high epithelial turnover rate [2]. It also has infiltrative propensities that lead to formation of daughter cysts and a high recurrence rate [3, 4]. For these reasons, OKC was revised to keratocystic odontogenic tumor (KCOT) in the 2005 WHO classification of head and neck tumours [5] to fit its biologic behavior.

KCOT typically occurs in the 2nd to 3rd decades of life with a slight male predilection [6]. KCOT growth is by increased epithelial turnover usually along the path of the least resistance and so cortical bony expansion is not common [7]. They are often asymptomatic [7]and, however, can present with a swelling, pain, paraesthesia, purulent discharge, nasal obstruction, and mobility of teeth [2]. If left untreated, KCOT can become quite large and locally destructive with invasion of adjacent structures [2].

KCOT lacks pathognomonic clinical and radiologic features and thus can mimic cystic, inflammatory, and neoplastic lesions affecting the jaw. These make clinical diagnosis challenging. The most common location in the jaw is the posterior mandible with occasional occurrence in the maxilla [8, 9]. Treatment of maxillary KCOT and subsequent rehabilitation is challenging because by the onset of symptoms it would have progressed widely beyond the confines of the maxilla. Treatment for KCOT ranges from conservative therapy such as marsupialization or enucleation (with or without cryotherapy or Carnoy's solution application) to marginal or radical resection [10, 11]. The aim of treatment should be eradication of the tumour, minimizing complications, and prevention of recurrence. When maxillary KCOT is extensive, a multidisciplinary approach with detailed treatment planning to salvage compromised vital and aesthetic structures is needed.

We therefore report the case of a massive bimaxillary KCOT which proved challenging in its clinical diagnosis and management.

## 2. Case Report

**Figure 1:**
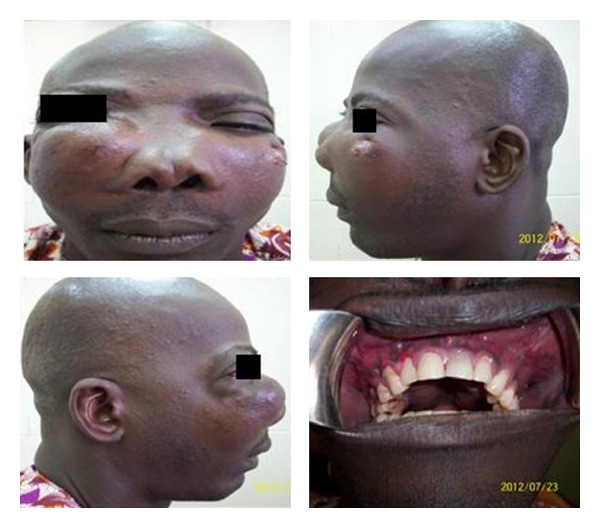
Clinical pictures showing a right and left maxillary swelling. The intraoral component involves both halves of the maxilla.

A 38-year-old male presented to our clinic on account of a diffuse bilateral maxillary swelling of 3 years duration. The swelling had progressively increased in size from a peanut-sized growth in the upper right quadrant to involve the right infraorbital region resulting in epiphora but without visual disturbances. Two years after the onset of the right maxillary swelling, he noticed a painless left maxillary swelling. The growth of both swellings resulted in mobility of adjacent teeth and recurrent infection evidenced by pus discharge. His medical history was noncontributory. Clinical examination revealed a nodular projection covered by darkly pigmented skin over the right maxillary swelling and a central area of ulceration over the left maxillary swelling (Figure 1). There was bilateral circumorbital edema and proptosis of both eyes, but vision was intact. The nasal bridge was flattened, the nasolabial folds were partially obliterated and both nostrils contained intranasal masses (Figure 1). Both swellings were firm in consistency but the entire mid-face was movable. Intraorally there was pus discharge from the socket of 15 and mobility of all the maxillary teeth.

**Figure 2:**
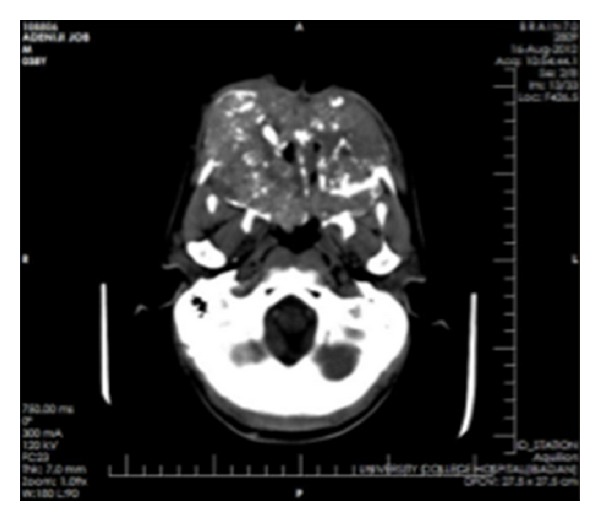
Axial computerized tomography showing a mass destroying the maxilla and the nasal tissues.

**Figure 3:**
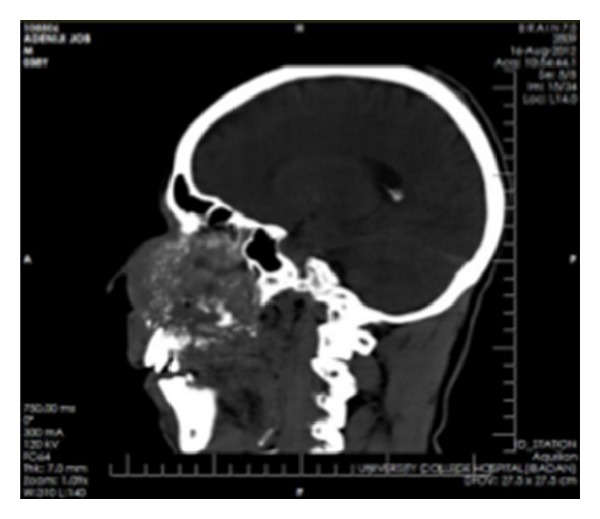
Sagittal computerized tomography (CT) showing destruction of the maxilla and nasal tissues with “floating” upper teeth.

**Figure 4:**
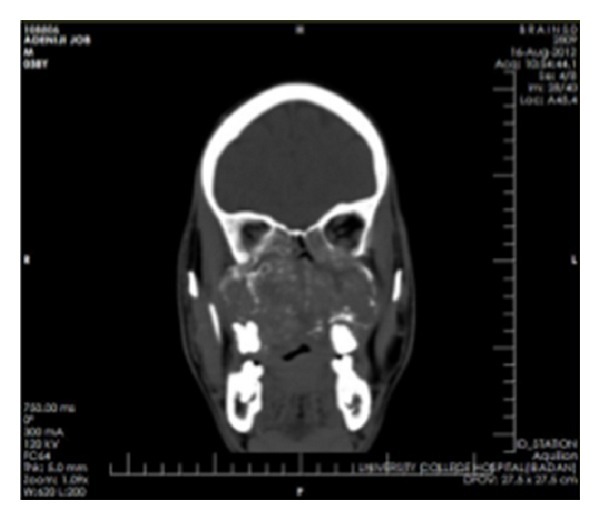
Coronal CT showing involvement of the paranasal sinuses and orbital floor.

**Figure 5:**
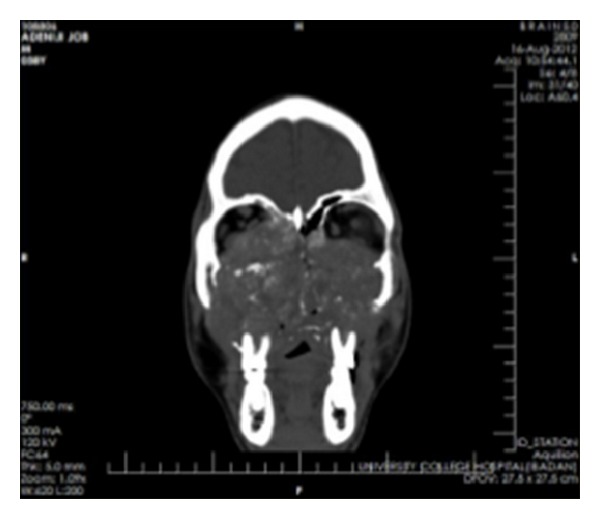
Coronal CT showing maxillary and orbital involvement.

**Figure 6:**
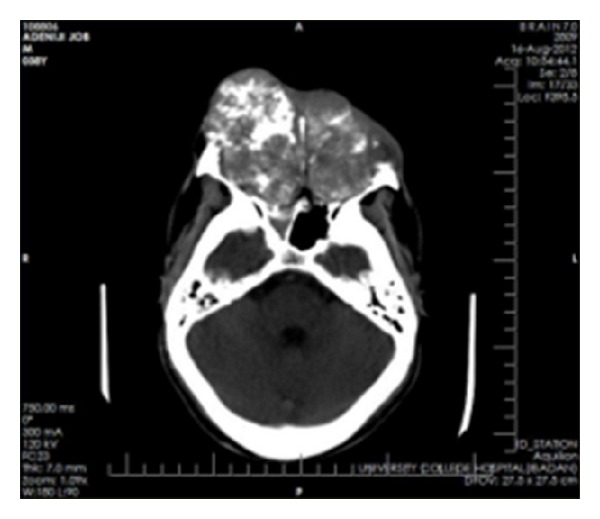
Axial CT showing the mass pushing out the orbital contents.

**Figure 7:**
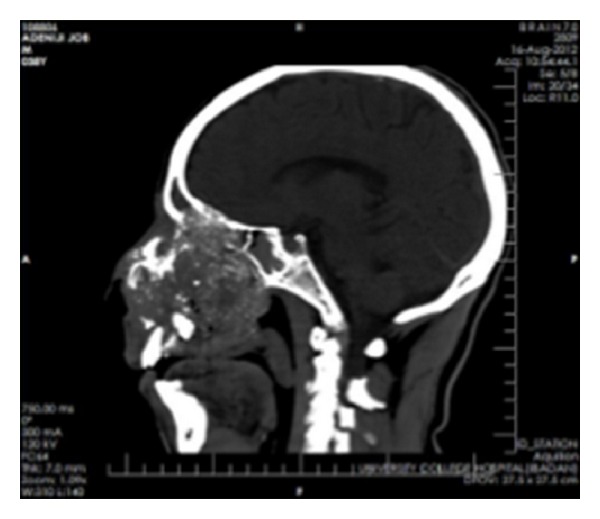
Sagittal CT showing involvement of the roof of the base of the skull.

**Figure 8:**
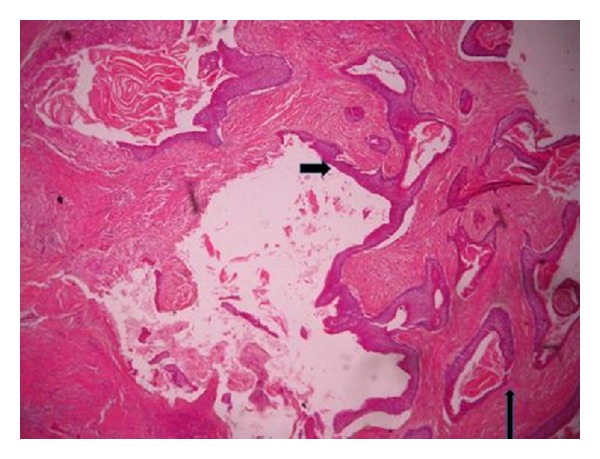
Photomicrograph showing a lining epithelium which is 6–8 cell layers thick, having basal cell palisading, surface parakeratinisation, and keratin squames within the lumen; there is artifactual separation of the lining epithelium from the connective tissue wall (short arrow). Also seen are other smaller cysts having a similar lining and containing keratin (long arrow) (×50).

**Figure 9:**
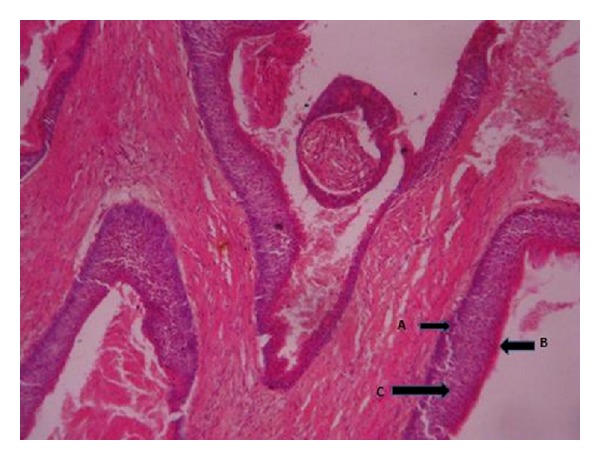
Photomicrograph of odontogenic keratocyst showing several daughter cysts. (A) Palisading of the basal cell layer. (B) Surface parakeratinisation. (C) Epithelial lining which is 6–8 cells thick (×100).

Clinical impressions were a deep mycotic infection, a non-Hodgkin's lymphoma, or an epithelial malignancy originating from the maxillary sinuses. Radiology, histology, and microbiology investigations were conducted. Craniofacial computerized tomography revealed complete destruction of the maxillae, palate, nasal septum, and nasal bone by a mass occupying the whole of the maxillae and maxillary antrum (Figures 2 and 3). There was destruction of the floor of the orbit, involvement of the extraocular muscles, and proptosis (Figures 4, 5, and 6). There was also destruction of the ethmoid, sphenoid, and frontal sinuses as well as the roof of the frontal sinus (Figure 7). The chest radiograph and microbiology findings were unremarkable. Histology showed multiple cysts that had lining epithelia 5–12 cells thick, having basal cell palisading and surface undulation with parakeratinization. Some of the cysts had keratin deposits within them (Figures 8 and 9). A diagnosis of KCOT was made.

## 3. Treatment

Surgical decompression was carried out under general anaesthesia using nasotracheal intubation. A modified Trotter-Weber Ferguson incision was made in the right maxillary region, while a Caldwell-Luc incision was made in the left maxilla to expose the lesion. The exposed cavities contained masses of friable necrotic tissue that were curetted and sent for histology. The cavities were well irrigated with normal saline and packed with dry sterile gauze. After shielding adjacent vital structures with Sofra-Tulle dressing, chemical cautery of the tumour bed was achieved with Carnoy's solution. The cavities were then packed with argotone soaked gauze to allow for proper haemostasis; this was left in place for 3 days. The patient had regular saline irrigation and dressing, initially every other day and after discharge on a weekly basis. Perceptible reduction in tumour size was achieved after a month of review, but complete resolution of the mass was not achieved before the patient defaulted from review visits and was lost to follow up.

## 4. Discussion

Appropriate therapy for any disease first requires correct diagnosis, which is formed by a combination of clinical details with or without supporting investigations. Our patient's pattern and site of presentation for KCOT were quite unusual, leading to a myriad of confusing clinical opinions and it was only following histologic examination that a working diagnosis of KCOT was reached. Unlike other jaw cysts with unremarkable histological features, the histology of the KCOT is rather unique and even though clinical features may not be clearly defined, the histology is distinctive. It is typified by a parakeratinized or orthokeratinized stratified squamous epithelial lining (6–8 cell layers thick) which is corrugated, a prominent and palisaded basal layer which may be cuboidal or columnar, lumen containing keratin, and a connective tissue wall without inflammation as well as absent rete pegs [12, 13]. The parakeratinized type is said to be commoner and tends to run a more aggressive course, while the orthokeratinized type has been suggested as a simple odontogenic keratocyst with no aggressive features [2, 13, 14]. The case reported here is a parakeratinized type. In reclassifying odontogenic keratocyst as a neoplasm, the budding of the basal layer into connective tissue wall is one of the factors considered important [3].

Although there is no universally accepted treatment yet for KCOT, the primary aim of treatment is to achieve total eradication utilizing an appropriate technique, taking into consideration site, size, location, invasion of the surrounding tissues, and previous treatments [15, 16]. Treatment options that have been used for KCOT include marsupialization, enucleation and curettage, and enucleation with chemical cautery (Carnoy's solution), thermal (cryotherapy), or mechanical (peripheral ostectomy) cautery of surrounding tissue and osseous resection with or without continuity defect. The extensive maxillary involvement of our patient necessitated a technique that would thoroughly remove tumour with the least compromise to function and aesthetics; hence the approach of combined surgical decompression, curettage, and chemical cautery.

Studies have shown that decompression and marsupialization relieves the pressure within the cavity of KCOT leading to a reduction in size and formation of new bone [17]. Following marsupialization for decompression, interleukin-1 and cytokeratin 10 which are important in cyst expansion are dissipated. Loss of these factors reduces the biologic aggressiveness of the tumour and subsequently the cystic lining is replaced by oral epithelium.

Recurrence rates following decompression and marsupialization of KCOT vary and recent studies have reported recurrence rates of 0%–18% [15, 18–20]. Surgical resection has the lowest recurrence rate with most authors reporting 0% recurrence, but it also has the highest morbidity and greatest challenge for reconstruction, aesthetics, and rehabilitation [21]. Recurrence following enucleation alone varies between 9% and 33% [16, 22, 23]. For our case, ablative surgery would not have removed the whole tumour and would also have created extensively mid-facial disfigurement, which would have been extremely difficult to reconstruct given our limited capabilities. We therefore opted for decompression and marsupialization with adjuvant Carnoy's solution cautery because of the site and size of tumour presented. It is documented that marsupialization for large KCOT when combined with adjuvant Carnoy's solution application gives good prognosis [15]. Drawbacks of this technique however include a need for repeated enucleation or subsequent resection, possibility of occult malignant transformation and the need for prolonged review visits. We assume that it was the periodic scheduled review visits that eventually led to our patient's weariness and eventual self-termination of his review visits. 

*Strength.* To the authors' knowledge this is the first case report, in the English literature, of a bimaxillary OKC that is not syndrome associated. Characteristically, multiple KCOT of the jaws is associated with the nevus basal cell carcinoma syndrome [24, 25]; our case however had no other lesions fitting the components of this syndrome. It is important therefore to report this case because of its oddity and perplexing clinical presentation.

*Limitation.* A more comprehensive follow-up process would have improved knowledge about the tumour biology and its response to the adopted treatment protocol, but the patient's right to decline sustained treatment is acknowledged.

## 5. Conclusion

The perplexing clinical presentation of a bimaxillary tumour and its challenging therapeutic intervention has been described for the benefit of awareness and early suspicion.
